# Cannabidivarin Treatment Ameliorates Autism-Like Behaviors and Restores Hippocampal Endocannabinoid System and Glia Alterations Induced by Prenatal Valproic Acid Exposure in Rats

**DOI:** 10.3389/fncel.2019.00367

**Published:** 2019-08-09

**Authors:** Erica Zamberletti, Marina Gabaglio, Marie Woolley-Roberts, Sharon Bingham, Tiziana Rubino, Daniela Parolaro

**Affiliations:** ^1^Department of Biotechnology and Life Sciences, University of Insubria, Varese, Italy; ^2^GW Research Ltd., Cambridge, United Kingdom; ^3^Zardi-Gori Foundation, Milan, Italy

**Keywords:** cannabidivarin, valproate, autism, endocannabinoid system, neuroinflammation

## Abstract

Autism spectrum disorder (ASD) is a developmental condition whose primary features include social communication and interaction impairments with restricted or repetitive motor movements. No approved treatment for the core symptoms is available and considerable research efforts aim at identifying effective therapeutic strategies. Emerging evidence suggests that altered endocannabinoid signaling and immune dysfunction might contribute to ASD pathogenesis. In this scenario, phytocannabinoids could hold great pharmacological potential due to their combined capacities to act either directly or indirectly on components of the endocannabinoid system and to modulate immune functions. Among all plant-cannabinoids, the phytocannabinoid cannabidivarin (CBDV) was recently shown to reduce motor impairments and cognitive deficits in animal models of Rett syndrome, a condition showing some degree of overlap with autism, raising the possibility that CBDV might have therapeutic potential in ASD. Here, we investigated the ability of CBDV treatment to reverse or prevent ASD-like behaviors in male rats prenatally exposed to valproic acid (VPA; 500 mg/kg i.p.; gestation day 12.5). The offspring received CBDV according to two different protocols: symptomatic (0.2/2/20/100 mg/kg i.p.; postnatal days 34–58) and preventative (2/20 mg/kg i.p.; postnatal days 19–32). The major efficacy of CBDV was observed at the dose of 20 mg/kg for both treatment schedules. CBDV in symptomatic rats recovered social impairments, social novelty preference, short-term memory deficits, repetitive behaviors and hyperlocomotion whereas preventative treatment reduced sociability and social novelty deficits, short-term memory impairments and hyperlocomotion, without affecting stereotypies. As dysregulations in the endocannabinoid system and neuroinflammatory markers contribute to the development of some ASD phenotypes in the VPA model, neurochemical studies were performed after symptomatic treatment to investigate possible CBDV’s effects on the endocannabinoid system, inflammatory markers and microglia activation in the hippocampus and prefrontal cortex. Prenatal VPA exposure increased CB1 receptor, FAAH and MAGL levels, enhanced GFAP, CD11b, and TNFα levels and triggered microglia activation restricted to the hippocampus. All these alterations were restored after CBDV treatment. These data provide preclinical evidence in support of the ability of CBDV to ameliorate behavioral abnormalities resembling core and associated symptoms of ASD. At the neurochemical level, symptomatic CBDV restores hippocampal endocannabinoid signaling and neuroinflammation induced by prenatal VPA exposure.

## Introduction

Autism spectrum disorder (ASD) represents a group of developmental disabilities whose primary symptoms include social communication and interaction impairments with restricted or repetitive motor movements, frequently associated with general cognitive deficits (American Psychiatric Association [APA], 2013). About 1% of the global population receives an ASD diagnosis (Baio et al., 2018), with a male to female ratio of 3:1 (Loomes et al., 2017). Diagnosis can be made as early as 2 years of age and patients are expected to have a normal lifespan. Despite the critical medical need, no approved treatments for the core symptoms of ASD are available; hence, reliable animal models are of fundamental importance for identifying and testing new therapeutic strategies. Although ASD is a typical human pathology, endophenotypes including impairments of social interaction, cognitive deficits, repetitive behaviors and motor dysfunctions can be reproduced in rodents by means of genetic and/or environmental manipulations (Ergaz et al., 2016; Kim et al., 2016). Environmentally based models are relevant when the same risk factor contributing to human autism produces similar brain and behavioral alterations in the animal. The use of valproic acid (VPA) in pregnancy has been consistently associated with an increased risk to develop congenital malformations and features of ASD in children (Duncan, 2007; Dufour-Rainfray et al., 2011; Christensen et al., 2013; Roullet et al., 2013; Ornoy et al., 2019). Similar to humans, rodents prenatally exposed to VPA show increased impaired social interactions and preference for social novelty, stereotypic repetitive behaviors, learning and memory defects and hypersensitivity (Schneider and Przewłocki, 2005; Dufour-Rainfray et al., 2010; Gandal et al., 2010; Kim et al., 2011; Mehta et al., 2011; Melancia et al., 2018). Therefore, because of its strong construct and face validity, the VPA animal model has been one of the most widely used to understand the neural underpinnings and to test novel therapeutic possibilities in the context of ASD (Mabunga et al., 2015; Ornoy et al., 2015; Tartaglione et al., 2019).

Recent studies in the prenatal VPA exposure model have implicated the endocannabinoid system in the development of ASD-like features. Changes in components of this neuromodulatory system were reported in different brain regions as a consequence of *in utero* VPA exposure, including alterations in 2-arachidonyilglycerol (2-AG) and anandamide (AEA) signaling and abnormalities in CB1 receptor (Kerr et al., 2013, 2016; Servadio et al., 2016; Melancia et al., 2018). Changes in other targets including PPARα, PPARγ, and GPR55 receptors were also observed (Kerr et al., 2013; Servadio et al., 2016; Melancia et al., 2018). Remarkably, recent studies have confirmed the presence of dysregulations of the endocannabinoid system in ASD patients (Brigida et al., 2017; Yeh and Levine, 2017; Karhson et al., 2018; Aran et al., 2019). A correlation between altered endocannabinoid signaling and ASD traits is supported by the observation that enhancing AEA signaling through inhibition of its degradation partially attenuated the behavioral phenotype induced by prenatal VPA exposure (Kerr et al., 2016; Servadio et al., 2016; Melancia et al., 2018), suggesting that modulation of the endocannabinoid signaling could represent a novel strategy for mitigating ASD symptoms. Besides the endocannabinoid system, recent evidence suggests that modulation of immune dysfunction might be beneficial toward ASD symptomatology. Indeed, signs of neuroinflammation have been reported in the brain of ASD patients, including microglia and astrocyte activation and increased expression of pro-inflammatory factors (Vargas et al., 2005; Morgan et al., 2010; Suzuki et al., 2013; Kern et al., 2016), reinforcing the idea that immunological dysfunction might play a role in ASD. In line with human evidence, signs of neuroinflammation, including increased reactive oxygen species, pro-inflammatory cytokines, astrocyte and microglia activation, have been observed in the VPA-induced ASD animal model (Lucchina and Depino, 2014; Codagnone et al., 2015; Deckmann et al., 2018; Kuo and Liu, 2018; Bronzuoli et al., 2019). The administration of compounds able to reduce this inflammatory response resulted in neuroprotection and amelioration of ASD-like phenotypes (Banji et al., 2011; Bambini-Junior et al., 2014; Pragnya et al., 2014; Al-Amin et al., 2015; Gao et al., 2016; Kumar and Sharma, 2016; Morakotsriwan et al., 2016; Bertolino et al., 2017; Deckmann et al., 2018; Fontes-Dutra et al., 2018), suggesting that inflammatory dysfunction might play a role in the development of ASD symptoms.

In this scenario, phytocannabinoids possess great and interesting pharmacological potentials. In addition to their indirect actions on components of the endocannabinoid system, plant-derived cannabinoids possess a broad range of pharmacological properties including proved anti-inflammatory and anti-oxidant properties (Nagarkatti et al., 2009; Ligresti et al., 2016; Morales et al., 2017; Maroon and Bost, 2018) that may contribute to achieve an overall beneficial effect in the context of ASD.

Recent studies have shown that the plant-derived cannabinoid Cannabidivarin (CBDV) exerts beneficial effects toward neurological and motor impairments as well as cognitive deficits in two animal models of Rett syndrome (Vigli et al., 2018; Zamberletti et al., 2019). CBDV’s simultaneous capacity to ameliorate neurological and motor defects as well as cognitive impairment in these animal models raises the possibility that this phytocannabinoid might have interesting yet unexplored therapeutic potential in ASD, prompting its evaluation in animal models of this disorder.

Therefore, in this study we examined the ability of CBDV treatment to reverse or prevent sociability and preference for social novelty deficits, repetitive behaviors, hyperactivity and recognition memory impairments in rats prenatally exposed to VPA (500 mg/kg i.p.; gestation day 12.5). To this aim, CBDV was administered using two treatment protocols in the male offspring of VPA-treated dams; a symptomatic treatment was performed between postnatal day (PND) 34–58 to assess the ability of CBDV to counteract VPA-induced autism-like behaviors whereas a preventative treatment was carried out from PND 19–32 to test CBDV’s ability to prevent the appearance of autism-like traits in the model. In addition to behavioral analysis, neurochemical studies were carried out after symptomatic CBDV treatment to investigate its effect on the endocannabinoid system as well as on inflammatory markers and microglia activation in the hippocampus and prefrontal cortex (PFC).

## Materials and Methods

### Prenatal VPA Administration

Pregnant Sprague-Dawley rats (Charles River, Calco, Italy) received a single intraperitoneal injection of sodium valproate 500 mg/kg (or saline) on gestation day (GD) 12.5. Sodium valproate (Sigma Aldrich, Milan) was dissolved in saline at a concentration of 250 mg/ml. Females were housed individually and were allowed to raise their own litters. Gross toxic effects were not observed in VPA-exposed rats in both dams and pups. No postnatal mortality was observed. Body weight was slightly but significantly reduced in VPA-exposed pups with respect to controls from PND 12–17. Eye opening was delayed in VPA-exposed pups with respect to vehicles between PND 14 and 16. At PND 9, male pups from VPA-treated dams showed significantly increased latencies in nest-seeking behavior compared to controls as well as impaired righting reflex (Supplementary Methods, Supplementary Results and Supplementary Figure S1). Nine vehicle- and 15 VPA-treated dams were used in this study. Litters were not culled and the offspring was weaned on PND 21, separated by sex and the animals were kept four to a cage, with controlled temperature and light conditions. Rats had free access to food (standard laboratory pellets) and water. All the experiments were performed in the light phase between 09:00 and 15:00. Experimental procedures were performed in accordance with the guidelines released by the Italian Ministry of Health (D.L. 2014/26) and the European Community directives regulating animal research (2010/63/EU). Protocols were approved by the Italian Minister for Scientific Research and all efforts were made to minimize the number of animals used and their suffering.

### CBDV Treatment

**FIGURE 1:**
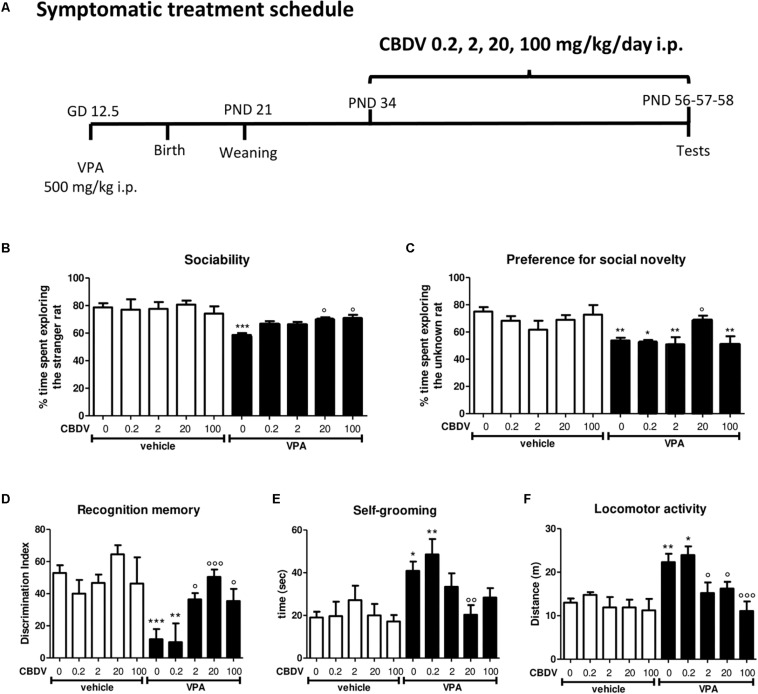
Symptomatic CBDV treatment protocol. Pregnant Sprague-Dawley rats received a single injection of VPA 500 mg/kg i.p. (or vehicle) at GD 12.5. **(A)** Symptomatic treatment with CBDV 0.2, 2, 20, 100 mg/kg/day i.p. was performed from PND 34 and male offspring was tested at PND 56 (three chamber test), 57 (NOR test), and 58 (repetitive behavior and locomotion). Effect of symptomatic CBDV 0.2, 2, 20, 100 mg/kg/day treatment in male offspring of VPA- and vehicle-exposed rats on **(B)** sociability and **(C)** social novelty preference as measured through the three chamber test; **(D)** short-term memory as measured through the novel object recognition; **(E)** compulsive self-grooming and **(F)** locomotor activity as measured in the activity cage. Data represent mean ± SEM of *n* = 9 vehicle-vehicle, *n* = 6 vehicle-CBDV 0.2 mg/kg, *n* = 6 vehicle-CBDV 2 mg/kg, *n* = 8 vehicle-CBDV 20 mg/kg, *n* = 6 vehicle-CBDV 100 mg/kg, *n* = 15 VPA-vehicle, *n* = 5 VPA-CBDV 0.2 mg/kg, *n* = 10 VPA-CBDV 2 mg/kg, *n* = 13 VPA-CBDV 20 mg/kg, *n* = 10 VPA-CBDV 100 mg/kg. Results were analyzed by two-way ANOVA followed by Tukey’s *post hoc* test (^∗∗∗^*p* < 0.001, ^∗∗^*p* < 0.01, ^∗^*p* < 0.05 vs. vehicle-vehicle;°°°*p* < 0.001,°°*p* < 0.01,°*p* < 0.05 vs. VPA-vehicle).

**FIGURE 2:**
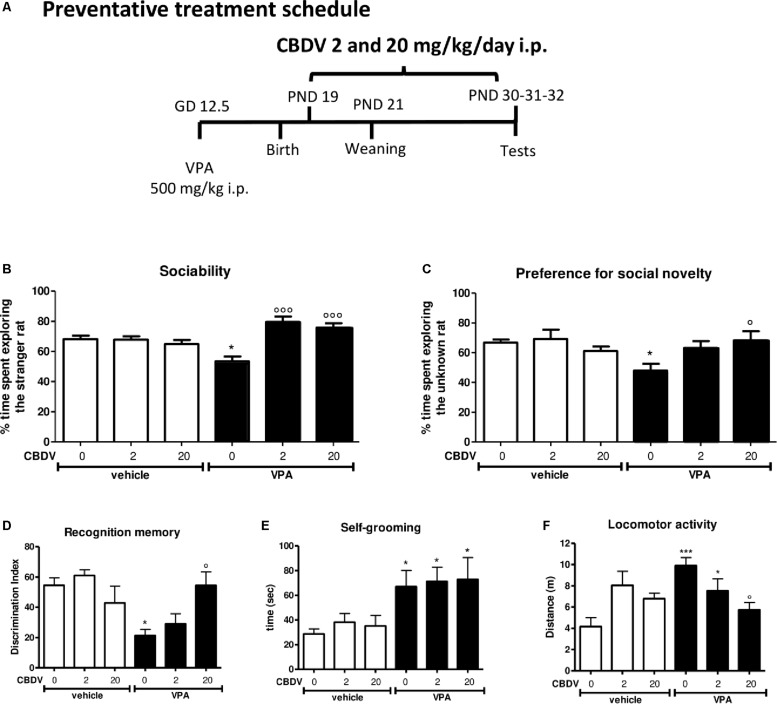
Preventative CBDV treatment protocol. Pregnant Sprague-Dawley rats received a single injection of VPA 500 mg/kg i.p. (or vehicle) at GD 12.5. **(A)** Preventative treatment with CBDV 2 or 20 mg/kg/day i.p. was performed from PND 19 and animals were tested at PND 30 (three chamber test), 31 (NOR test), and 32 (repetitive behavior and locomotion). Effect of preventative CBDV 2, 20 mg/kg/day treatment in male offspring of VPA- and vehicle-exposed rats on **(B)** sociability and **(C)** social novelty preference as measured through the three chamber test; **(D)** short-term memory as measured through the novel object recognition test; **(E)** compulsive self-grooming and **(F)** locomotor activity as measured in the activity cage. Data represent mean ± SEM of *n* = 7 vehicle-vehicle, *n* = 6 vehicle-CBDV 2 mg/kg, *n* = 6 vehicle-CBDV 20 mg/kg, *n* = 6 VPA-vehicle, *n* = 8 VPA-CBDV 2 mg/kg, *n* = 8 VPA-CBDV 20 mg/kg and were analyzed using two-way ANOVA followed by Tukey’s *post hoc* test (^∗∗∗^*p* < 0.001, ^∗^*p* < 0.05 vs. vehicle-vehicle;°°°*p* < 0.01,°*p* < 0.05 vs. VPA-vehicle).

A total of 54 rats from 9 vehicle-treated dams and 75 rats from 15 VPA-treated dams were used in this study. Pharmacological treatments and behavioral testing were carried out in 5–6 rats per litter during four separate experiments. Pure CBDV was provided by GW Research Ltd. (Cambridge, United Kingdom) and dissolved in ethanol, Kolliphor EL and saline (2:1:18) and was administered according to the treatment schedules reported in Figures 1A, 2A. Symptomatic treatment with CBDV 0.2, 2, 20 or 100 mg/kg/day i.p. was performed starting from PND 34 (early adolescence) to 58 (early adulthood) in the male offspring of dams injected with VPA 500 mg/kg (or saline) on GD 12.5 (Figure 1A). Starting from PND 56, a series of behavioral tests was performed. The three chamber test was carried out at PND 56 in order to assess the effect of chronic CBDV on sociability and social novelty preference, the novel object recognition (NOR) test was performed at PND 57 to assess short-term memory, and locomotion and stereotyped/repetitive behaviors were measured in the activity cage at PND 58. Preventative treatment with CBDV 2 or 20 mg/kg/day i.p. was performed starting from PND 19 (pre-weaning) to 32 (post-weaning) in the male offspring of VPA- and vehicle-treated dams (Figure 2A). Behavioral analysis was carried out at PND 30 (three chamber test), 31 (NOR test), and 32 (repetitive behavior and locomotion).

### Behavioral Studies

#### Three-Chamber Test

The three-chamber test was performed to measure social approach and social preference. In brief, animals were placed into a novel arena (80 cm × 31.5 cm × 40 cm) composed of three communicating chambers separated by Perspex walls with central openings allowing access to all chambers for 5 min. Distance moved (meters) and time spent (seconds) in the various compartments were recorded during this time to evaluate general locomotor activity and ensure that animals did not have a preference for a particular side of the arena. Following this acclimatization period, animals were briefly confined to the central chamber while an unfamiliar rat confined in a small wire cage was placed in one of the outer chambers. An identical empty wire cage was placed in the other chamber. The unfamiliar rat was randomly either assigned to the right or left chamber of the arena. The test animal was then allowed to explore the arena for a further 5 min. The arena was cleaned between animals with 0.1% acetic acid. Time spent engaging in investigatory behavior with the rat was evaluated with the aid of ANY-maze program (Ugo Basile, Italy) in order to examine social approach. To investigate the preference for social novelty, a novel unfamiliar rat was then placed in the empty cage and the test animal was allowed to explore the arena for a further 5 min. Time spent engaging in investigatory behavior with the novel unfamiliar rat was evaluated with the aid of Anymaze program (Ugo Basile, Italy) in order to examine the preference for social novelty. Sociability index and preference for social novelty index were calculated as the ratio of time spent exploring the stranger rat (sociability) or the unknown rat (preference for novelty) vs. the total time of exploration × 100.

#### Novel Object Recognition (NOR) Test

The experimental apparatus used for the object recognition test was an open-field box (43 cm × 43 cm × 32 cm) made of Plexiglas, placed in a dimly illuminated room. The experiment was performed and analyzed as previously described (Zamberletti et al., 2014). Animals performed each test individually. Briefly, each animal was placed in the arena and allowed to explore two identical previously unseen objects for 5 min (familiarization phase). After an inter-trial interval of 3 min one of the two familiar objects was replaced by a novel, previously unseen object and rats were returned to the arena for the 5-min test phase. The arena was cleaned between animals with 0.1% acetic acid. During the test phase the time spent exploring the familiar object (Ef) and the new object (En) was videotaped and recorded separately by two observers blind to the treatment groups and the discrimination index was calculated as follows: [(En − Ef)/(En + Ef)] × 100.

#### Activity Cage

Locomotor activity was recorded in an activity cage (40 cm × 40 cm × 40 cm) for 20 min with the aid of Anymaze program (Ugo Basile, Italy). In this period, repetitive behaviors (compulsive self-grooming) were measured by an observer blind to the treatment group. The cage was cleaned between animals with 0.1% acetic acid.

### Biochemical Studies

All animals underwent behavioral assessment. 24 h after the last CBDV (or vehicle) injection, all the animals were euthanized, their brain tissues collected and randomly assigned to different procedures for subsequent biochemical analysis. For Western blot analysis, PFC and hippocampi were dissected, frozen in liquid nitrogen and stored at −80°C; for immunohistochemistry, the brains were quickly removed and post-fixed in 4% paraformaldehyde in 100 mM phosphate buffer pH7.4, stored in fixative for 48 h, kept in 30% sucrose for 24 h. Coronal sections were serially collected using a Leica cryostat CM1510 set to 40 μm thickness and a −20°C chamber temperature.

#### Western Blotting

Cytosolic fractions from rat hippocampus and PFC were obtained using a protocol published by Shen and Chen (2013), with slight modifications. In brief, animals were sacrificed and cerebral areas quickly dissected. Samples were homogenized by 25 strokes in a glass-glass homogenizer in 0.32 M sucrose solution containing 20 mM HEPES, 1 mM MgCl_2_, protease inhibition cocktail, and 0.1 mM phenylmethylsulfonyl fluoride (PMSF) (pH 7.4). The homogenized tissue was centrifuged at 500 × *g* for 2 min. Resultant pellets (P1) were resuspended in 500 μL of a solution containing HEPES 20 mM, MgCl_2_ 1.5 mM, NaCl 420 mM, EDTA 0.2 mM, glycerol 25%, DTT 2 mM, PMSF 2 mM, protease inhibition cocktail and stored as nuclear fraction. The resulting supernatant (S1) was centrifuged at 10,000 × *g* for 10 min to obtain a fraction containing mitochondria and synaptosome-enriched pellets (P2) and the supernatant (S2) containing soluble proteins. S2 fraction was conserved as cytosolic fraction while the P2 fraction was resuspended in 0.32 M sucrose, layered onto 0.8 M sucrose and centrifuged at 4100 rpm for 15 min in a swinging bucket rotor to obtain crude synaptosome fractions. The protein concentrations were determined according to the Micro-BCA assay kit (Pierce, Rockford, IL, United States).

Equal amount of protein lysates from the cytosolic fractions (30 μg) were run on a 10% SDS-polyacrylamide gel. The proteins were then transferred to polyvinylidene difluoride (PVDF) membranes, blocked for 2 h at room temperature in 5% dry skimmed milk in TBS 1×, 0.1% tween-20 before incubation overnight at 4°C with the primary antibody. The following primary antibodies were used: rabbit polyclonal anti-CB1 (1:1000; Cayman Chemical, United States), rabbit polyclonal anti-CB2 (1:1000; Cayman Chemical, United States), rabbit polyclonal anti-FAAH (1:1000; Cayman Chemical, United States), rabbit polyclonal anti-MAGL (1:1000; Cayman Chemical, United States), rabbit polyclonal anti-NAPE-PLD (1:3000; Cayman Chemical, United States), goat polyclonal anti-DAGLα (1:1000; AbCam, United Kingdom), rabbit polyclonal anti-GFAP (1:1000; Sigma Aldrich, United States), rabbit polyclonal anti-CD11b (1:1000; Novus Biologicals, United States), rabbit polyclonal anti-TNF-α (1:2000; Millipore, United States).

Bound antibodies were detected with horseradish peroxidase (HRP) conjugated secondary anti-rabbit, anti-mouse or anti-goat antibodies (1:1000–10000; Santa Cruz Biotechnology, United States) for 1 h at room temperature and visualized using ECL Western Blotting Detection Reagents (Bio-Rad Laboratories, Hercules, CA, United States). For detection of β-actin, the blots were stripped with Restore Western Blot Stripping Buffer (Thermo Scientific, Rockford, IL, United States) and re-blotted with mouse monoclonal anti-β-actin (1:20000; Sigma Aldrich, United States) overnight at 4°C and visualized as described above. Bands were detected with G-Box (Syngene) instrument. For densitometry, images were digitally scanned and optical density of the bands was quantified using ImageJ software (NIH, Bethesda, MD, United States) and normalized to controls. To allow comparison between different blots, the density of the bands was expressed as arbitrary units.

#### Immunohistochemistry

Free-floating sections containing the dorsal hippocampus were washed three times in 0.05% Tryton X-100 in TBS, incubated with 3% normal goat serum, 0.05% Triton X-100 in TBS for 1 h at room temperature and then overnight at 4°C with rabbit anti-IBA1 antibody (1:1000, Wako, Neuss, Germany) diluted in blocking solution. After blocking peroxidase activity with 0.3% H_2_O_2_ in TBS for 15 min, sections were washed in TBS and incubated for 4 h at room temperature with HRP-conjugated goat anti-rabbit antibody (1:500; Santa Cruz Biotechnology, United States). The peroxidase activity was revealed with 0.05% diaminobenzidine and 0.03% hydrogen peroxide in PBS for 10 min. After several washes in PBS, sections were mounted on gelatin-coated slides, dehydrated and cover slipped. For each animal, a complete series of one-in-six sections (240 μm apart) through the hippocampus was analyzed. Digital Images were captured using Retiga R1 CCD camera (QImaging, Surrey, BC, Canada) attached to an Olympus BX51 (Tokyo, Japan) polarizing/light microscope. Ocular imaging software (QImaging) was used to import images from the camera. Images of microglia cells in the subgranular zone of the hippocampus were acquired by first delineating the brain sections and the regions of interest at low magnification (×4 objective) and the region of interest outlines were further refined under a ×40 objective. Three mice per each experimental group (four sections/mouse) were analyzed. The morphometric analysis was carried out in DAB-stained microglial cells labeled with IBA-1 antibody. For this purpose, cells were selected and cropped according to the following criteria: (i) random selection in the subgranular zone of the hippocampus; (ii) no overlapping with neighboring cells; and (iii) complete soma and branches (at least apparently). Selection was done blinded to the treatment. Eight cells from each animal were analyzed. Each grayscale single cell cropped image was processed in a systematic way to obtain binary image using the same threshold for all pictures. The binary image was edited to clear the background and transformed into a filled shape and its pairwise outline shape that were used for morphological parameters measurements. Analysis was performed using FIJI free software (NIH, Bethesda, MD, United States). Four parameters, measured on the filled and outlined processed images obtained as described previously (Fernández-Arjona et al., 2017), were analyzed: cell area, cell perimeter, roundness of the soma and soma area.

### Statistical Analysis

The Shapiro–Wilk normality test was first used to determine if the data were normally distributed. Results were then expressed as mean ± SEM and quantitative normally distributed data were analyzed by two-way ANOVA (VPA and CBDV as independent variables), followed by Tukey’s *post hoc* test. The level of statistical significance was set at *p* < 0.05.

## Results

### Behavioral Studies

#### Symptomatic CBDV Treatment

Figure 1 represents the effect of symptomatic CBDV treatment (0.2, 2, 20, and 100 mg/kg/day; PND 34–58; Figure 1A) on autism-like phenotypes in the male offspring of VPA-exposed rats.

##### Sociability and social novelty preference

In the three-chamber test (Figures 1B,C), no differences in the time spent in each compartment of the apparatus were observed during the habituation phase, suggesting that animals belonging to all the experimental groups did not show a preference for a particular side of the arena (data not shown). During the sociability test (Figure 1B), two-way ANOVA revealed significant main effects of VPA [*F*_(1,75)_ = 34.35; *p* < 0.0001] and VPA × CBDV interaction [*F*_(4,75)_ = 2.636; *p* = 0.0405] on the percentage of time spent exploring the stranger rat with respect to the empty cage. Indeed, the percentage of time spent by male VPA-exposed rats in the chamber containing the unfamiliar rat was significantly reduced compared to controls (58.673 ± 1.389% in VPA-vehicle vs. 78.668 ± 3.069% in vehicle-vehicle). CBDV at all doses tested did not affect sociability when administered to control animals. CBDV treatment at doses of 20 and 100 mg/kg significantly restored the impairment in sociability observed in VPA rats while doses of 0.2 and 2 mg/kg failed to reverse the sociability deficit in VPA-treated rats.

Concerning the preference for social novelty (Figure 1C), significant effects of VPA [*F*_(1,75)_ = 21.54; *p* < 0.0001] and VPA × CBDV interaction [*F*_(4,75)_ = 2.556; *p* = 0.0456] were observed on the percentage of time spent exploring the unknown rat during the test. Control male rats spent a significantly higher percentage of time exploring the novel rat than the known rat (75.021 ± 3.301%). In contrast, VPA animals spent a similar time exploring the two stimuli (53.757 ± 2.072%). Treatment with CBDV 20 mg/kg completely reversed the deficit in social preference in VPA rats, as demonstrated by the fact that VPA-CBDV rats spent significantly more time exploring the novel rats with respect to the familiar one (69.128 ± 2.900%). In contrast, CBDV 0.2, 2, and 100 mg/kg were not able to restore social novelty preference in VPA rats. None of the doses tested had *per se* any effect on social novelty preference when administered to controls.

##### Short-term recognition memory

Figure 1D represents the effect of chronic CBDV 0.2, 2, 20, and 100 mg/kg/day treatment on short-term memory as evaluated through the NOR test. Total exploration time during the familiarization phase was similar in all the groups under investigation (data not shown). During the test phase, significant effects of VPA [*F*_(1,75)_ = 17.47; *p* < 0.0001] and VPA × CBDV interaction [*F*_(4,75)_ = 4.565; *p* = 0.0024] were observed. Prenatal VPA administration significantly impaired short-term memory, as demonstrated by a significant reduction of the discrimination index by about 81% with respect to controls. Statistical analysis did not reveal any significant effect of CBDV when administered in control rats. Interestingly, CBDV 2, 20, and 100 mg/kg significantly reversed the short-term memory deficit in male VPA rats, without affecting *per se* recognition memory when administered to vehicles. In contrast, CBDV 0.2 mg/kg failed to counteract memory deficits in VPA rats, the discrimination index being still reduced by about 79% with respect to controls.

##### Compulsive self-grooming and locomotion

The effect of chronic CBDV 0.2, 2, 20, and 100 mg/kg/day treatment on compulsive self-grooming and locomotor activity is reported in Figures 1E,F respectively.

Statistical analysis showed significant effects of VPA [*F*_(1,75)_ = 17.06; *p* < 0.0001] and VPA × CBDV interaction [*F*_(4,75)_ = 2.518; *p* = 0.0483] and a trend for CBDV’s effect [*F*_(4,75)_ = 2.381; *p* = 0.0590] on self-grooming. VPA exposure significantly increased the time spent by male rats in compulsive self-grooming by about 121% with respect to vehicles. Chronic CBDV administration at the dose of 20 mg/kg significantly normalized the time spent in repetitive behaviors in VPA-treated rats without having any effect when administered to controls. CBDV 2 and 100 mg/kg showed a trend to ameliorate compulsive self-grooming in VPA-vehicle rats. The lowest dose of CBDV was instead ineffective.

Main effects of VPA [*F*_(1,75)_ = 17.52; *p* < 0.0001], CBDV [*F*_(4,75)_ = 5.527; *p* = 0.0006] and VPA × CBDV interaction [*F*_(4,75)_ = 2.485; *p* = 0.0500] were also found on locomotor activity. Indeed, VPA administration significantly increased locomotion by about 69% compared to controls and CBDV administration at the dose of 2, 20, and 100 mg/kg significantly normalized it. In contrast, CBDV 0.2 mg/kg failed to recover hyperlocomotion in VPA-exposed rats. None of the CBDV doses tested affected locomotion in control animals.

#### Preventative CBDV Treatment

Figure 2 represents the effect of preventative CBDV treatment (2 and 20 mg/kg/day; PND 19–32; Figure 2A) on autism-like phenotypes in the male offspring of VPA-exposed rats.

##### Sociability and social novelty preference

The effect of preventative CBDV 2 and 20 mg/kg/day treatment on sociability in the male offspring of VPA- and vehicle-exposed rats, as measured through the three chamber apparatus is shown in Figure 2B. During the habituation phase, VPA and CBDV administration did not affect the time spent in each compartment of the maze and all animals spent similar amounts of time exploring each compartment of the apparatus (data not shown). During the sociability test, significant effects of VPA [*F*_(1,35)_ = 9.385; *p* = 0.0005] and VPA × CBDV interaction [*F*_(2,35)_ = 11.78; *p* = 0.0001] were found. Vehicle-vehicle rats spent significantly more time exploring the unfamiliar rat compared to the empty cage (68.113 ± 2.345%). A similar effect was also observed in vehicle animals treated with CBDV 2 mg/kg (67.773 ± 2.297%) and 20 mg/kg (64.861 ± 2.788%). In contrast, male VPA-exposed rats spent similar amount of time in the chamber containing the unfamiliar rat compared to the time spent in the empty compartment when compared to controls (53.484 ± 3.220%), indicating a deficit in sociability. Chronic CBDV treatment at both doses significantly prevented the deficit in sociability in VPA rats without affecting sociability in control animals.

In the preference for social novelty trial (Figure 2C), statistical analysis showed a significant VPA × CBDV interaction [*F*_(2,35)_ = 4.426; *p* = 0.0196] and a trend for VPA [*F*_(1,35)_ = 4.088; *p* = 0.0511] and CBDV [*F*_(2,35)_ = 3.174; *p* = 0.0545] effects. Control male rats spent a significantly greater percentage of time exploring the novel rat than the known rat (66.819 ± 1,995%). A similar effect was also present in vehicle animals treated with CBDV 2 mg/kg (69.199 ± 6.227%) and 20 mg/kg (61.095 ± 3.0147%). Conversely, VPA animals spent a similar percentage of time exploring the two stimuli (48.003 ± 4.535%). Treatment with CBDV 2 and 20 mg/kg significantly prevented the deficit in social novelty preference in VPA-exposed rats.

##### Short-term recognition memory

Figure 2D depicts the effect of preventative CBDV 2 and 20 mg/kg/day treatment on short-term memory in vehicle- and VPA-treated rats evaluated through the NOR test. Total exploration time during the familiarization phase was similar in all the groups under investigation (data not shown).

Two-way ANOVA revealed significant effects of VPA [*F*_(1,35)_ = 8.943; *p* = 0.0051] and VPA × CBDV interaction [*F*_(2,35)_ = 6.127; *p* = 0.0052] on short-term memory. Prenatal VPA administration significantly impaired short-term memory, as demonstrated by a significant reduction of the discrimination index by about 60.9% with respect to controls. Both doses of CBDV did not affect recognition memory when administered to vehicles. CBDV 20 mg/kg significantly prevented the short-term memory deficit in male VPA rats, whereas the lowest dose was ineffective.

##### Compulsive self-grooming and locomotion

Figures 2E,F represent the effect of preventative CBDV 2 and 20 mg/kg/day treatment on repetitive behaviors (compulsive self-grooming) and locomotion, respectively.

A significant main effect of VPA [*F*_(1,35)_ = 13.64; *p* = 0.0008] was found on self-grooming behavior. Indeed, VPA exposure significantly increased the time spent by male rats in compulsive self-grooming by about 133.9% with respect to vehicle-vehicle animals. CBDV treatment did not affect self-grooming in control rats and no dose of CBDV tested was able to prevent compulsive self-grooming in male VPA-exposed rats.

Significant VPA [*F*_(1,35)_ = 7.301; *p* = 0.0106] and VPA × CBDV [*F*_(2,35)_ = 7.554; *p* = 0.0019] effects were observed on locomotor activity in male rats. VPA administration significantly increased locomotor activity by about 137.9% compared to controls. Chronic CBDV treatment did not affect locomotion when administered to controls while CBDV administration in VPA rats significantly prevented hyperlocomotion only at the dose of 20 mg/kg.

### Biochemistry

All biochemical studies were performed 24 h after the last CBDV (or vehicle) injection using the dose of CBDV more efficacious toward ASD-like phenotypes (i.e., 20 mg/kg).

#### Effect of Symptomatic CBDV Treatment (20 mg/kg) on Components of the Endocannabinoid System in the Hippocampus of Vehicle- and VPA-Exposed Rats

**FIGURE 3:**
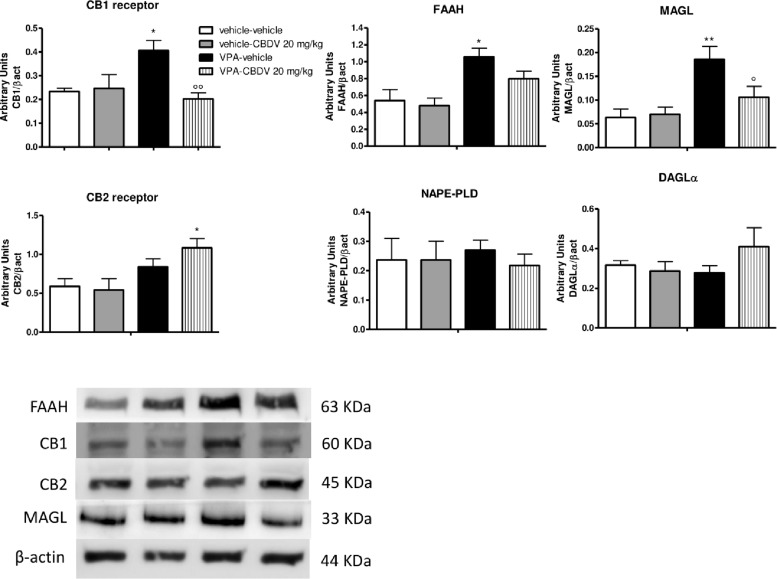
Effect of symptomatic CBDV 20 mg/kg/day treatment on components of the endocannabinoid system in the hippocampus of the male offspring of VPA- and vehicle-exposed rats as measured by means of Western blot analysis in cytosolic fractions. Data represent mean ± SEM of *n* = 3 vehicle-vehicle, *n* = 3 vehicle-CBDV 20 mg/kg, *n* = 5 VPA-vehicle, *n* = 5 VPA-CBDV 20 mg/kg and were analyzed using two-way ANOVA followed by Tukey’s *post hoc* test (^∗∗^*p* < 0.01, ^∗^*p* < 0.05 vs. vehicle-vehicle;°°*p* < 0.01,°*p* < 0.05 vs. VPA-vehicle).

Figure 3 shows the effects of CBDV on the protein levels of components of the endocannabinoid system in the hippocampus of vehicle and VPA rats. Two-way ANOVA revealed significant effects of VPA [*F*_(1,12)_ = 8.198; *p* = 0.0143], CBDV [*F*_(1,12)_ = 13.32; *p* = 0.0033], and VPA × CBDV interaction [*F*_(1,12)_ = 15.88; *p* = 0.0018] on CB1 receptor expression. Prenatal VPA exposure significantly increased CB1 receptor levels in the hippocampus compared to vehicle littermates. CBDV treatment at the behaviorally efficacious dose of 20 mg/kg significantly normalized CB1 receptor expression without affecting its levels when administered to vehicles. A significant effect of VPA [*F*_(1,12)_ = 10.31; *p* = 0.0075] was also found on CB2 receptor levels. Indeed, prenatal VPA exposure alone did not affect receptor expression whereas a significant increase in CB2 receptor levels was observed in VPA-exposed rats after chronic administration of CBDV 20 mg/kg. Statistical analysis revealed significant effects of VPA [*F*_(1,12)_ = 8.722; *p* = 0.0121] and VPA × CBDV interaction [*F*_(1,12)_ = 5.830; *p* = 0.0326] on FAAH protein levels within the hippocampus. FAAH expression was significantly enhanced in VPA exposed rats and it was reduced by chronic CBDV treatment. A similar effect was also found regarding MAGL expression. Indeed, MAGL levels were increased by prenatal VPA exposure and CBDV administration significantly restored MAGL expression in the hippocampus of VPA exposed rats without affecting its levels when administered in controls [VPA: *F*_(1,12)_ = 7.536; *p* = 0.0178; VPA × CBDV interaction: *F*_(1,12)_ = 4.499; *p* = 0.0564]. Neither VPA exposure nor CBDV treatment alone or in combination affected NAPE-PLD and DAGLα expression in this brain region.

#### Effect of Symptomatic CBDV Treatment (20 mg/kg) on Neuroinflammatory Markers and Microglia Morphology in the Hippocampus of Vehicle- and VPA-Exposed Rats

**FIGURE 4:**
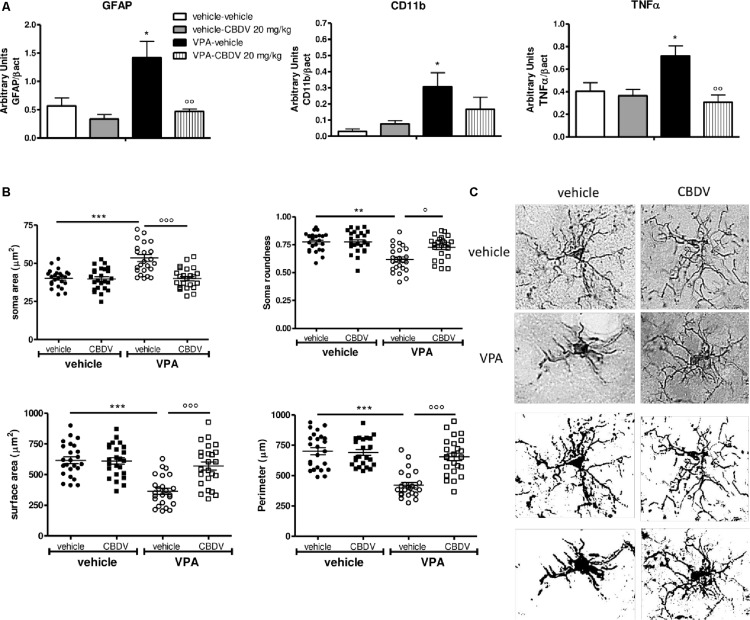
**(A)** Effects of prenatal VPA exposure and symptomatic CBDV 20 mg/kg/day treatment on the expression of the astrocyte marker GFAP, the microglia marker CD11b and the pro-inflammatory cytokine TNF-α in the hippocampus as measured by means of Western blot analysis in cytosolic fractions. Data represent mean ± SEM of *n* = 3 vehicle-vehicle, *n* = 3 vehicle-CBDV 20 mg/kg, *n* = 5 VPA-vehicle, *n* = 5 VPA-CBDV 20 mg/kg and were analyzed using two-way ANOVA followed by Tukey *post hoc* test. **(B)** Effect of prenatal VPA exposure and CBDV treatment (20 mg/kg/day; PND 34–58) on microglia morphology in the hippocampus as analyzed through Iba1 immunostaining. **(C)** Representative Iba-1 staining and microglia morphology at ×40 magnification (upper panels: grayscale images; lower panels: filled images). Data represent mean ± SEM of three animals per group (4 slices/animal, 240 μm apart; 8 cells/animals) and were analyzed using two-way ANOVA followed by Tukey’s *post hoc* test (^∗∗∗^*p* < 0.001,^∗∗^*p* < 0.01, ^∗^*p* < 0.05 vs. vehicle-vehicle;°°°*p* < 0.001,°°*p* < 0.01,°*p* < 0.05 vs. VPA-vehicle).

Figure 4A shows the effects of prenatal VPA exposure and symptomatic CBDV treatment on the expression of the astrocyte marker GFAP, the microglia marker CD11b and the pro-inflammatory cytokine TNF-α in the hippocampus. Two-way ANOVA showed significant effects of VPA [*F*_(1,12)_ = 12.12; *p* = 0.0045], CBDV [*F*_(1,12)_ = 14.71; *p* = 0.0024] and VPA × CBDV interaction [*F*_(1,12)_ = 8.361; *p* = 0.0135] on GFAP expression. Prenatal VPA exposure significantly increased GFAP protein levels in the hippocampus. Symptomatic CBDV treatment completely restore GFAP expression in VPA rats without affecting the levels of this marker in control animals. Similarly, a significant increase in CD11b expression was found in the hippocampus after VPA exposure *in utero* and CBDV administration showed a trend toward reducing the expression of this marker when given to VPA rats without having any effect *per se* in control animals [VPA: *F*_(1,12)_ = 5.673; *p* = 0.0308]. Significant effects of VPA [*F*_(1,12)_ = 4.902; *p* = 0.0469] and VPA × CBDV interaction [*F*_(1,12)_ = 8.531; *p* = 0.0128] were observed on TNF-α, whose expression was significantly increased in the hippocampus of VPA pre-treated rats. CBDV treatment significantly restored TNF-α levels when chronically administered to VPA rats.

Figures 4B,C shows the effects of prenatal VPA and symptomatic CBDV treatments on some parameters of microglia morphology, namely soma size and roundness as well as surface area and perimeter. Statistical analysis revealed main effects of VPA [*F*_(1–8)_ = 17.55; *p* = 0.0030], CBDV [*F*_(1–8)_ = 18.41; *p* = 0.0026], and VPA × CBDV interaction [*F*_(1–8)_ = 16.26; *p* = 0.0038] on soma size. Soma size was significantly increased in rats prenatally exposed to VPA compared to controls. CBDV treatment did not alter soma size in control animals but it significantly restored soma area when administered to VPA-treated rats. Similarly, main effects of VPA [*F*_(1–8)_ = 25.21; *p* = 0.0010], CBDV [*F*_(1–8)_ = 7.376; *p* = 0.0264], and VPA × CBDV interaction [*F*_(1–8)_ = 7.221; *p* = 0.0276] were observed on soma roundness. Prenatal VPA exposure significantly reduced roundness of the soma of Iba-1 positive cells in the hippocampus with respect to controls. CBDV completely rescued this alteration without affecting soma roundness in control rats.

Statistical analysis also revealed main effects of VPA [*F*_(1–8)_ = 26.18; *p* = 0.0009], CBDV [*F*_(1–8)_ = 11.98; *p* = 0.0086], and VPA × CBDV interaction [*F*_(1–8)_ = 13.60; *p* = 0.0062] on surface area and perimeter [VPA: *F*_(1–8)_ = 34.68; *p* = 0.0004, CBDV: *F*_(1–8)_ = 17.28; *p* = 0.0032, and VPA × CBDV interaction: *F*_(1–8)_ = 20.71; *p* = 0.0019]. Morphological analysis showed that prenatal VPA exposure significantly reduced both surface area and perimeter of microglia cells. Again, CBDV did not affect either surface area or perimeter of Iba-1 positive cells in control animals but its administration significantly normalized both parameters in rats prenatally exposed to VPA.

#### Effect of Symptomatic CBDV Treatment (20 mg/kg) on Components of the Endocannabinoid System and Neuroinflammatory Markers in the PFC of Vehicle- and VPA-Exposed Rats

**FIGURE 5:**
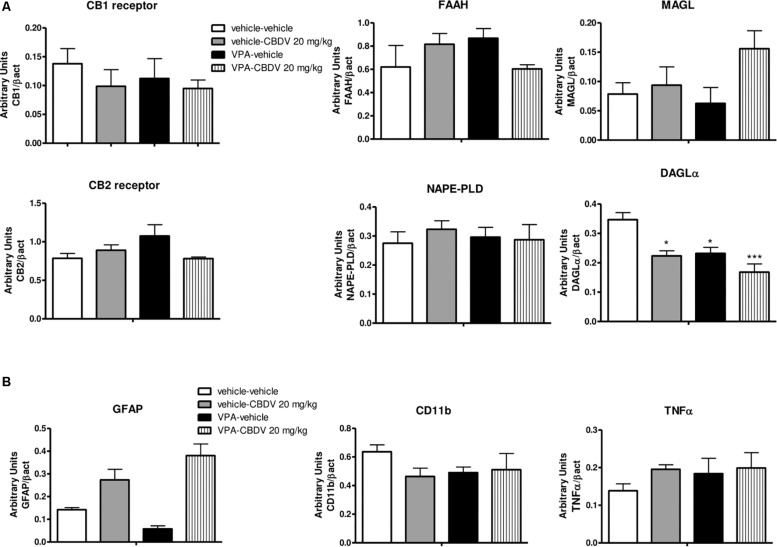
Effect of prenatal VPA exposure and symptomatic CBDV 20 mg/kg/day treatment on protein levels of **(A)** components of the endocannabinoid system and **(B)** the astrocyte marker GFAP, the microglia marker CD11b and the pro-inflammatory cytokine TNF-α in the PFC as measured by means of Western blot analysis in cytosolic fractions. Data represent mean ± SEM of *n* = 3 vehicle-vehicle, *n* = 3 vehicle-CBDV 20 mg/kg, *n* = 5 VPA-vehicle, *n* = 5 VPA-CBDV 20 mg/kg and were analyzed using two-way ANOVA followed by Tukey’s *post hoc* test. (^∗∗^*p* < 0.01, ^∗^*p* < 0.05 vs. vehicle-vehicle).

As shown in Figure 5A, no effect of prenatal VPA and symptomatic CBDV treatments were found on CB1 and CB2 receptor, FAAH, MAGL, and NAPE-PLD levels in the PFC. In contrast, statistical analysis revealed significant effects of VPA [*F*_(1,12)_ = 11.02; *p* = 0.0041] and CBDV [*F*_(1,12)_ = 13.39; *p* = 0.0033] on DAGLα expression. Prenatal VPA exposure significantly reduced DAGLα levels in this brain area and a similar effect was observed after CBDV administration both in control and in VPA rats. Concerning the neuroinflammatory markers (Figure 5B), neither VPA nor CBDV affected GFAP, CD11b, and TNF-α levels in the PFC.

## Discussion

This study was performed to determine whether CBDV treatment could be beneficial toward ASD-like features induced by prenatal VPA exposure in rats. In particular, we evaluated CBDV’s efficacy toward VPA-induced deficits in sociability and social novelty preference, repetitive self-grooming behavior, recognition memory deficits and hyperactivity in the male offspring of VPA-treated dams using either symptomatic (PND 34–58) and preventative (PND 19–32) treatment protocols.

Results here presented show that chronic CBDV treatment was able to ameliorate ASD-like signs induced by prenatal VPA exposure in the male offspring. Treatment with CBDV at the dose of 20 mg/kg in symptomatic rats rescued deficits in sociability and social novelty preference, repetitive self-grooming, recognition memory impairment and hyperactivity. In contrast, doses of 2 and 100 mg/kg only partially affected the phenotypes under investigation: CBDV 2 mg/kg recovered short-term memory deficits and hyperlocomotion while, at the dose of 100 mg/kg, CBDV’s activity against the deficit in social novelty preference and stereotyped behaviors was lost. The lowest dose tested, 0.2 mg/kg, was instead ineffective. Hence, in this experimental model and at the doses tested in this study, CBDV does not show a linear dose-response curve, being more effective at the intermediate dose of 20 mg/kg with respect to doses of 2 and 100 mg/kg. This could suggest that CBDV might display a bell shaped dose-response curve as demonstrated for other phytocannabinoids in some animal models (Pertwee, 2004; Mishima et al., 2005; Mechoulam et al., 2007; Campos and Guimarães, 2008; Zuardi, 2008; Zanelati et al., 2010; Campos et al., 2012). Alternatively, it could be possible that the experimental paradigm used in our study failed to identify a linear dose response range.

Slightly different results were found in the preventative treatment schedule. Preventative CBDV treatment at the dose of 2 mg/kg significantly prevented sociability and social novelty preference deficits but failed to ameliorate repetitive behaviors, hyperactivity and short-term memory deficits. In contrast, CBDV 20 mg/kg prevented sociability and social novelty preference deficits, normalized locomotor activity and improved short-term memory deficits but was ineffective toward repetitive self-grooming behavior.

An aspect that emerges from these data is the different efficacy of CBDV depending on the time of administration. In fact, while symptomatic treatment at the dose of 20 mg/kg appears to be efficacious in reverting most ASD-like phenotypes, none of CBDV’s doses tested in this study completely prevented the behaviors under investigation when administered during early developmental period (i.e., peri-weaning). Nevertheless, independent from the time window of administration, CBDV at all doses was devoid of any side effect when administered to control animals, further supporting the safety profile of this compound (Huizenga et al., 2019).

In the search for possible correlates of the effects observed at the behavioral level, we performed neurochemical analysis in the PFC and hippocampus of VPA-exposed rats treated with CBDV at the dose that showed the maximum behavioral efficacy, i.e., 20 mg/kg. Neurochemical investigations were carried out after symptomatic CBDV treatment only, as the translational value of a preventative treatment in the context of ASD is quite limited at present. In fact, diagnosis of ASD is based on the identification of symptoms and a preventative treatment could only be useful when reliable biomarkers are available. Identifying biomarkers for early disease detection, especially in high-risk populations, is therefore a primary need to allow for earlier pharmacological interventions, with the intent of improving outcomes. Of note, results here presented raise the intriguing possibility that early CBDV treatment might partially prevent/attenuate the development of ASD symptoms.

Recent data from human and animal studies suggest an involvement of the endocannabinoid system in the pathogenesis of ASD. Lower circulating endocannabinoid levels and changes of endocannabinoid receptors and enzymes have been reported in ASD patients (Siniscalco et al., 2013, 2014; Brigida et al., 2017; Karhson et al., 2018; Aran et al., 2019). Animal studies support human data demonstrating the presence of alterations in several components of the endocannabinoid system in the brain of both genetic and environmental ASD models (Maccarrone et al., 2010; Jung et al., 2012; Foldy et al., 2013; Kerr et al., 2013; Speed et al., 2015; Zamberletti et al., 2017). Remarkably, pharmacological modulation of the endocannabinoid signaling can ameliorate some ASD-like phenotypes in animals (Busquets-Garcia et al., 2013; Qin et al., 2015; Gomis-González et al., 2016; Kerr et al., 2016; Servadio et al., 2016; Wei et al., 2016; Melancia et al., 2018), suggesting that interfering with the endocannabinoid system might be beneficial for relieving ASD symptomatology. In line with literature data, we found that prenatal VPA exposure triggers endocannabinoid system alterations in the brain of the male offspring. These changes are more pronounced in the hippocampus with respect to the PFC. Specifically, enhanced FAAH and MAGL expression together with an up-regulation of CB1 receptor were observed in the hippocampus while reduced DAGLα levels were detected in the PFC. Increases in the enzymes responsible for AEA and 2-AG degradation in the hippocampus and the reduction of 2-AG synthesis in the PFC possibly support the presence of a reduced endocannabinoid tone in the brain of VPA-exposed animals, in line with previous findings (Kerr et al., 2013). Interestingly, FAAH, MAGL, and CB1 receptor protein levels returned to control level following CBDV treatment, suggesting that CBDV’s ability to restore endocannabinoid system abnormalities might contribute to its beneficial effects on ASD-like behaviors. In addition, we found that CBDV treatment also up-regulated CB2 receptor expression in the hippocampus of VPA-exposed rats. CB2 is emerging as an important regulator of the inflammatory response in the central nervous system (Basu and Dittel, 2011). Although still debated, several authors suggest its immunosuppressive and neuroprotective potential (Ehrhart et al., 2005; Palazuelos et al., 2009; Tumati et al., 2012; Zoppi et al., 2014; Navarro et al., 2016). Its deletion in animals usually exacerbates the inflammatory phenotype in several models and CB2 activation by cannabinoids can slow the progression of some diseases, in addition to reducing inflammation (Turcotte et al., 2016), suggesting that modulation of CB2 receptor could be beneficial for relieving inflammation. Although our data does not allow to establish a causal relationship, we observed that CBDV-induced up-regulation of hippocampal CB2 receptor was associated with the rescue of the neuroinflammatory markers GFAP, CD11b, and TNF-α in the same brain region. Further supporting its either direct or indirect neuroprotective effects, we observed that CBDV treatment can restore microglia activation and consecutive morphological changes in terms of cell size and soma shape in the hippocampus of VPA-treated animals. Hence, CBDV treatment restores the endocannabinoid system and reduces neuroinflammation in the VPA model but, based on the present data, we cannot establish any causality between the two events. Although literature data clearly indicate that alterations of the endocannabinoid system and neuroinflammation co-exist in the brain of VPA-treated rats, there is no evidence about a possible correlation between the two events in the animal model at baseline. Indeed, the consequences of a modulation of either the endocannabinoid system or inflammation have been evaluated in the VPA model but no study has checked whether modulating one of the two events affects the other. Starting from the observation that CBDV does not directly interact with the cannabinoid system at physiologically relevant concentration, we speculate that restoration of the homeostatic endocannabinoid tone by CBDV might be secondary to its effect on neuroinflammation. We hypothesize that CBDV might promote a shift from a pro-inflammatory state, also called the “M1 phenotype,” presenting neurotoxic activities and releasing pro-inflammatory signals, to a more neuroprotective profile called the “M2 phenotype” which involves anti-inflammatory responses. Of note, upregulation of CB2 receptors has been associated with a restoration of tissue homeostasis in pathological neuroinflammatory conditions (Miller and Devi, 2011) and our observation that CBDV increases the expression of CB2 receptors in VPA rats further supports its anti-inflammatory action in this model. We speculate that microglia cells shifted to an anti-inflammatory phenotype would then increase endocannabinoid production (Mecha et al., 2015), which by acting autocrinally and/or paracrinally could facilitate/amplify the M2 anti-inflammatory phenotype and might contribute to the restoration of endocannabinoid signaling.

## Conclusion

This study provides preclinical evidence in support of the ability of CBDV to ameliorate behavioral abnormalities resembling the core and associated symptoms of ASD, a developmental condition for which no cure is available. Restoration of hippocampal endocannabinoid signaling and neuroinflammation are likely to contribute to CBDV’s beneficial effects toward ASD-like phenotypes induced by prenatal VPA exposure.

Although further work is required to determine the mechanism(s) of action of CBDV and to evaluate its effect in other animal models, the present results identify for the first time CBDV as a suggested candidate for the treatment of ASD.

## Data Availability

The raw data supporting the conclusions of this manuscript will be made available on request, without undue reservation, to any qualified researcher.

## Author Contributions

EZ performed the histochemical and Western blot experiments and wrote the first draft of the manuscript. MG performed the pharmacological treatments and behavioral experiments. MW-R and SB helped with the manuscript writing and English editing. TR helped with the data interpretation and manuscript writing. DP conceived the study, supervised the project, helped with the data interpretation and manuscript writing. All authors contributed and have approved the final draft of the manuscript.

## Conflict of Interest Statement

DP and TR received research grants from GW Research Ltd. MW-R and SB are employees of GW Research Ltd. The remaining authors declare that the research was conducted in the absence of any commercial or financial relationships that could be construed as a potential conflict of interest.

## References

[B1] Al-Amin M. M. Rahman M. M. Khan F. R. Zaman F. Mahmud Reza H. (2015). Astaxanthin improves behavioral disorder and oxidative stress in prenatal valproic acid-induced mice model of autism. *Behav. Brain Res.* 286 112–121. 10.1016/j.bbr.2015.02.041 25732953

[B2] American Psychiatric Association [APA] (2013). *Diagnostic and Statistical Manual of Mental Disorders*, 5th Edn Washington, DC: American Psychiatric Publishing, Inc.

[B3] Aran A. Eylon M. Harel M. Polianski L. Nemirovski A. Tepper S. (2019). Lower circulating endocannabinoid levels in children with autism spectrum disorder. *Mol. Autism.* 10:2. 10.1186/s13229-019-0256-6 30728928 PMC6354384

[B4] Baio J. Wiggins L. Christensen D. L. Maenner M. J. Daniels J. Warren Z. (2018). Prevalence of autism spectrum disorder among children aged 8 years – autism and developmental disabilities monitoring network, 11 sites, United States, 2014. *MMWR Surveill. Summ.* 67 1–23. 10.15585/mmwr.ss6706a1 29701730 PMC5919599

[B5] Bambini-Junior V. Zanatta G. Della Flora Nunes G. Mueller, de Melo G. Michels M. (2014). Resveratrol prevents social deficits in animal model of autism induced by valproic acid. *Neurosci. Lett.* 583 176–181. 10.1016/j.neulet.2014.09.039 25263788

[B6] Banji D. Banji O. J. Abbagoni S. Hayath M. S. Kambam S. Chiluka V. L. (2011). Amelioration of behavioral aberrations and oxidative markers by green tea extract in valproate induced autism in animals. *Brain Res.* 1410 141–151. 10.1016/j.brainres.2011.06.063 21820650

[B7] Basu S. Dittel B. N. (2011). Unraveling the complexities of cannabinoid receptor 2 (CB2) immune regulation in health and disease. *Immunol. Res.* 51 26–38. 10.1007/s12026-011-8210-5 21626285 PMC4624216

[B8] Bertolino B. Crupi R. Impellizzeri D. Bruschetta G. Cordaro M. Siracusa R. (2017). Beneficial effects of co-ultramicronized palmitoylethanolamide/luteolin in a mouse model of autism and in a case report of autism. *CNS Neurosci. Ther.* 23 87–98. 10.1111/cns.12648 27701827 PMC6492645

[B9] Brigida A. L. Schultz S. Cascone M. Antonucci N. Siniscalco D. (2017). Endocannabinod signal dysregulation in autism spectrum disorders: a correlation link between inflammatory state and neuro-immune alterations. *Int. J. Mol. Sci.* 18:E1425. 10.3390/ijms18071425 28671614 PMC5535916

[B10] Bronzuoli M. R. Facchinetti R. Ingrassia D. Sarvadio M. Schiavi S. Steardo L. (2019). Neuroglia in the autistic brain: evidence from a preclinical model. *Mol. Autism* 9:66. 10.1186/s13229-018-0254-0 30603062 PMC6307226

[B11] Busquets-Garcia A. Gomis-Gonzalez M. Guegan T. Agustin-Pavon C. Pastor A. Mato S. (2013). Targeting the endocannabinoid system in the treatment of fragile X syndrome. *Nat. Med.* 19 603–607. 10.1038/nm.3127 23542787

[B12] Campos A. C. Guimarães F. S. (2008). Involvement of 5HT1A receptors in the anxiolytic-like effects of cannabidiol injected into the dorsolateral periaqueductal gray of rats. *Psychopharmacology* 199 223–230. 10.1007/s00213-008-1168-x 18446323

[B13] Campos A. C. Moreira F. A. Gomes F. V. Del Bel E. A. Guimarães F. S. (2012). Multiple mechanisms involved in the large-spectrum therapeutic potential of cannabidiol in psychiatric disorders. *Philos. Trans. R. Soc. Lond. B Biol. Sci.* 367 3364–3378. 10.1098/rstb.2011.0389 23108553 PMC3481531

[B14] Christensen J. Grønborg T. K. Sørensen M. J. Schendel D. Parner E. T. Pedersen L. H. (2013). Prenatal valproate exposure and risk of autism spectrum disorders and childhood autism. *JAMA* 309 1696–1703. 10.1001/jama.2013.2270 23613074 PMC4511955

[B15] Codagnone M. G. Podestá M. F. Uccelli N. A. Reinés A. (2015). Differential local connectivity and neuroinflammation profiles in the medial prefrontal cortex and hippocampus in the valproic acid rat model of autism. *Dev. Neurosci.* 37 215–231. 10.1159/000375489 25895486

[B16] Deckmann I. Schwingel G. B. Fontes-Dutra M. Bambini-Junior V. Gottfried C. (2018). Neuroimmune alterations in autism: a translational analysis focusing on the animal model of autism induced by prenatal exposure to valproic acid. *Neuroimmunomodulation* 25 285–299. 10.1159/000492113 30157484

[B17] Dufour-Rainfray D. Vourc’h P. Le Guisquet A. M. Garreau L. Ternant D. Bodard S. (2010). Behavior and serotonergic disorders in rats exposed prenatally to valproate: a model for autism. *Neurosci. Lett.* 470 55–59. 10.1016/j.neulet.2009.12.054 20036713

[B18] Dufour-Rainfray D. Vourc’h P. Tourlet S. Guilloteau D. Chalon S. res C. R. (2011). Fetal exposure to teratogens: evidence of genes involved in autism. *Neurosci. Biobehav. Rev.* 35 1254–1265. 10.1016/j.neubiorev.2010.12.013 21195109

[B19] Duncan S. (2007). Teratogenesis of sodium valproate. *Curr. Opin. Neurol.* 20 175–180. 10.1097/WCO.0b013e32805866fb 17351488

[B20] Ehrhart J. Obregon D. Mori T. Hou H. Sun N. Bai Y. (2005). Stimulation of cannabinoid receptor 2 (CB2) suppresses microglial activation. *J. Neuroinflammation* 2:29. 10.1186/1742-2094-2-29 16343349 PMC1352348

[B21] Ergaz Z. Weinstein-Fudim L. Ornoy A. (2016). Genetic and non-genetic animal models for autism spectrum disorders (ASD). *Reprod Toxicol.* 64 116–140. 10.1016/j.reprotox.2016.04.024 27142188

[B22] Fernández-Arjona M. D. M. Grondona J. M. Granados-Durán P. Fernández-Llebrez P. López-Ávalos M. D. (2017). Microglia morphological categorization in a rat model of neuroinflammation by hierarchical cluster and principal components analysis. *Front. Cell Neurosci.* 11:235. 10.3389/fncel.2017.00235 28848398 PMC5550745

[B23] Foldy C. Malenka R. C. Sudhof T. C. (2013). Autism-associated neuroligin-3 mutations commonly disrupt tonic endocannabinoid signaling. *Neuron* 78 498–509. 10.1016/j.neuron.2013.02.036 23583622 PMC3663050

[B24] Fontes-Dutra M. Santos-Terra J. Deckmann I. Brum Schwingel G. Della-Flora Nunes G. Hirsch M. M. (2018). resveratrol prevents cellular and behavioral sensory alterations in the animal model of autism induced by valproic acid. *Front. Synaptic. Neurosci.* 10:9. 10.3389/fnsyn.2018.00009 29872390 PMC5972198

[B25] Gandal M. J. Edgar J. C. Ehrlichman R. S. Mehta M. Roberts T. P. Siegel S. J. (2010). Validating γ oscillations and delayed auditory responses as translational biomarkers of autism. *Biol. Psychiatry* 68 1100–1106. 10.1016/j.biopsych.2010.09.031 21130222 PMC5070466

[B26] Gao J. Wang X. Sun H. Cao Y. Liang S. Wang H. (2016). Neuroprotective effects of docosahexaenoic acid on hippocampal cell death and learning and memory impairments in a valproic acid-induced rat autism model. *Int. J. Dev. Neurosci.* 49 67–78. 10.1016/j.ijdevneu.2015.11.006 26639559

[B27] Gomis-González M. Busquets-Garcia A. Matute C. Maldonado R. Mato S. Ozaita A. (2016). Possible therapeutic doses of cannabinoid type 1 receptor antagonist reverses key alterations in fragile X syndrome mouse model. *Genes* 7:E56. 10.3390/genes7090056 27589806 PMC5042387

[B28] Huizenga M. N. Sepulveda-Rodriguez A. Forcelli P. A. (2019). Preclinical safety and efficacy of cannabidivarin for early life seizures. *Neuropharmacology* 148 189–198. 10.1016/j.neuropharm.2019.01.002 30633929 PMC6424614

[B29] Jung K. M. Sepers M. Henstridge C. M. Lassalle O. Neuhofer D. Martin H. (2012). Uncoupling of the endocannabinoid signalling complex in a mouse model of fragile X syndrome. *Nat. Commun.* 3:1080. 10.1038/ncomms2045 23011134 PMC3657999

[B30] Karhson D. S. Krasinska K. M. Dallaire J. A. Libove R. A. Phillips J. M. Chien A. S. (2018). Plasma anandamide concentrations are lower in children with autism spectrum disorder. *Mol Autism.* 9:18. 10.1186/s13229-018-0203-y 29564080 PMC5848550

[B31] Kern J. K. Geier D. A. Sykes L. K. Geier M. R. (2016). Relevance of neuroinflammation and encephalitis in autism. *Front. Cell. Neurosci.* 9:519 10.3389/fncel.2015.00519 PMC4717322 26834565

[B32] Kerr D. M. Downey L. Conboy M. Finn D. P. Roche M. (2013). Alterations in the endocannabinoid system in the rat valproic acid model of autism. *Behav. Brain Res.* 249 124–132. 10.1016/j.bbr.2013.04.043 23643692

[B33] Kerr D. M. Gilmartin A. Roche M. (2016). Pharmacological inhibition of fatty acid amide hydrolase attenuates social behavioural deficits in male rats prenatally exposed to valproic acid. *Pharmacol. Res.* 113 228–235. 10.1016/j.phrs.2016.08.033 27592249

[B34] Kim K. C. Gonzales E. L. Lázaro M. T. Choi C. S. Bahn G. H. Yoo H. J. (2016). Clinical and neurobiological relevance of current animal models of autism spectrum disorders. *Biomol Ther.* 24 207–243. 10.4062/biomolther.2016.061 27133257 PMC4859786

[B35] Kim K. C. Kim P. Go H. S. Choi C. S. Yang S. I. Cheong J. H. (2011). The critical period of valproate exposure to induce autistic symptoms in sprague-dawley rats. *Toxicol Lett.* 201 137–142. 10.1016/j.toxlet.2010.12.018 21195144

[B36] Kumar H. Sharma B. (2016). Minocycline ameliorates prenatal valproic acid induced autistic behaviour, biochemistry and blood brain barrier impairments in rats. *Brain Res.* 1630 83–97. 10.1016/j.brainres.2015.10.052 26551768

[B37] Kuo H. Y. Liu F. C. (2018). molecular pathology and pharmacological treatment of autism spectrum disorder-like phenotypes using rodent models. *Front. Cell Neurosci.* 12:422. 10.3389/fncel.2018.00422 30524240 PMC6262306

[B38] Ligresti A. De Petrocellis L. Di Marzo V. (2016). From phytocannabinoids to cannabinoid receptors and endocannabinoids: pleiotropic physiological and pathological roles through complex pharmacology. *Physiol. Rev.* 96 1593–1659. 10.1152/physrev.00002.2016 27630175

[B39] Loomes R. Hull L. Mandy W. P. L. (2017). What Is the Male-to-female ratio in autism spectrum disorder? A systematic review and meta-analysis. *J. Am. Acad. Child Adolesc. Psychiatry* 56 466–474. 10.1016/j.jaac.2017.03.013 28545751

[B40] Lucchina L. Depino A. M. (2014). Altered peripheral and central inflammatory responses in a mouse model of autism. *Autism Res.* 7 273–289. 10.1002/aur.1338 24124122

[B41] Mabunga D. F. Gonzales E. L. Kim J. W. Kim K. C. Shin C. Y. (2015). Exploring the validity of valproic acid animal model of autism. *Exp. Neurobiol.* 24 285–300. 10.5607/en.2015.24.4.285 26713077 PMC4688329

[B42] Maccarrone M. Rossi S. Bari M. de Chiara V. Rapino C. Musella A. (2010). Abnormal mGlu 5 receptor/endocannabinoid coupling in mice lacking FMRP and BC1 RNA. *Neuropsychopharmacology* 35 1500–1509. 10.1038/npp.2010.19 20393458 PMC3055456

[B43] Maroon J. Bost J. (2018). Review of the neurological benefits of phytocannabinoids. *Surg. Neurol. Int.* 9:91. 10.4103/sni.sni_45_18 29770251 PMC5938896

[B44] Mecha M. Feliú A. Carrillo-Salinas F. J. Rueda-Zubiaurre A. Ortega-Gutiérrez S. de Sola R. G. (2015). Endocannabinoids drive the acquisition of an alternative phenotype in microglia. *Brain Behav. Immun.* 49 233–245. 10.1016/j.bbi.2015.06.002 26086345

[B45] Mechoulam R. Peters M. Murillo-Rodriguez E. Hanus L. O. (2007). Cannabidiol–recent advances. *Chem. Biodivers.* 4 1678–1692. 10.1002/cbdv.200790147 17712814

[B46] Mehta M. V. Gandal M. J. Siegel S. J. (2011). mGluR5-antagonist mediated reversal of elevated stereotyped, repetitive behaviors in the VPA model of autism. *PLoS One* 6:e26077. 10.1371/journal.pone.0026077 22016815 PMC3189241

[B47] Melancia F. Schiavi S. Servadio M. Cartocci V. Campolongo P. Palmery M. (2018). Sex-specific autistic endophenotypes induced by prenatal exposure to valproic acid involve anandamide signalling. *Br. J. Pharmacol.* 175 3699–3712. 10.1111/bph.14435 29968249 PMC6109221

[B48] Miller L. K. Devi L. A. (2011). the highs and lows of cannabinoid receptor expression in disease: mechanisms and their therapeutic implications. *Pharmacol. Rev.* 63 461–470. 10.1124/pr.110.003491 21752875 PMC3141881

[B49] Mishima K. Hayakawa K. Abe K. Ikeda T. Egashira N. Iwasaki K. (2005). Cannabidiol prevents cerebral infarction via a serotonergic 5-hydroxytryptamine1A receptor-dependent mechanism. *Stroke* 36 1077–1082. 10.1161/01.STR.0000163083.59201.34 15845890

[B50] Morakotsriwan N. Wattanathorn J. Kirisattayakul W. Chaisiwamongkol K. (2016). Autistic-like behaviors, oxidative stress status, and histopathological changes in cerebellum of valproic acid rat model of autism are improved by the combined extract of purple rice and silkworm pupae. *Oxid. Med. Cell. Longev.* 2016:3206561. 10.1155/2016/3206561 27034733 PMC4806649

[B51] Morales P. Hurst D. P. Reggio P. H. (2017). Molecular targets of the phytocannabinoids: a complex picture. *Prog. Chem. Org. Nat. Prod.* 103 103–131. 10.1007/978-3-319-45541-9_4 28120232 PMC5345356

[B52] Morgan J. T. Chana G. Pardo C. A. Achim C. Semendeferi K. Buckwalter J. (2010). Microglial activation and increased microglial density observed in the dorsolateral prefrontal cortex in autism. *Biol. Psychiatry* 68 368–376. 10.1016/j.biopsych.2010.05.024 20674603

[B53] Nagarkatti P. Pandey R. Rieder S. A. Hegde V. L. Nagarkatti M. (2009). Cannabinoids as novel anti-inflammatory drugs. *Future Med. Chem.* 1 1333–1349. 10.4155/fmc.09.93 20191092 PMC2828614

[B54] Navarro G. Morales P. Rodríguez-Cueto C. Fernández-Ruiz J. Jagerovic N. Franco R. (2016). Targeting cannabinoid cb2 receptors in the central nervous system. medicinal chemistry approaches with focus on neurodegenerative disorders. *Front. Neurosci.* 10:406. 10.3389/fnins.2016.00406 27679556 PMC5020102

[B55] Ornoy A. Weinstein-Fudim L. Ergaz Z. (2015). Prenatal factors associated with autism spectrum disorder (ASD). *Reprod Toxicol.* 56 155–169. 10.1016/j.reprotox.2015.05.007 26021712

[B56] Ornoy A. Weinstein-Fudim L. Ergaz Z. (2019). Prevention or amelioration of autism-like symptoms in animal models: will it bring us closer to treating human ASD? *Int. J. Mol. Sci.* 20 E1074. 10.3390/ijms20051074 30832249 PMC6429371

[B57] Palazuelos J. Aguado T. Pazos M. R. Julien B. Carrasco C. Resel E. (2009). Microglial CB2 cannabinoid receptors are neuroprotective in Huntington’s disease excitotoxicity. *Brain* 132(Pt 11), 3152–3164. 10.1093/brain/awp23 19805493

[B58] Pertwee R. G. (2004). “The pharmacology and therapeutic potential of cannabidiol,” in *Cannabinoids*, ed. Di Marzo V. (Kluwer: Academic/Plenum Publishers), 32–83.

[B59] Pragnya B. Kameshwari J. Veeresh B. (2014). Ameliorating effect of piperine on behavioral abnormalities and oxidative markers in sodium valproate induced autism in BALB/C mice. *Behav. Brain Res.* 270 86–94. 10.1016/j.bbr.2014.04.045 24803211

[B60] Qin M. Zeidler Z. Moulton K. Krych L. Xia Z. Smith C. B. (2015). Endocannabinoid-mediated improvement on a test of aversive memory in a mouse model of fragile X syndrome. *Behav. Brain Res.* 291 164–171. 10.1016/j.bbr.2015.05.003 25979787 PMC5003021

[B61] Roullet F. I. Lai J. K. Foster J. A. (2013). In utero exposure to valproic acid and autism-a current review of clinical and animal studies. *Neurotoxicol Teratol.* 36 47–56. 10.1016/j.ntt.2013.01.004 23395807

[B62] Schneider T. Przewłocki R. (2005). Behavioral alterations in rats prenatally exposed to valproic acid: animal model of autism. *Neuropsychopharmacology* 30 80–89. 10.1038/sj.npp.1300518 15238991

[B63] Servadio M. Melancia F. Manduca A. di Masi A. Schiavi S. Cartocci V. (2016). Targeting anandamide metabolism rescues core and associated autistic-like symptoms in rats prenatally exposed to valproic acid. *Transl. Psychiatry* 6:e902. 10.1038/tp.2016.182 27676443 PMC5048215

[B64] Shen C. Chen Y. (2013). Preparation of pre- and post-synaptic density fraction from mouse cortex. *Bio Protoc.* 3:e880 10.21769/BioProtoc.880

[B65] Siniscalco D. Bradstreet J. J. Cirillo A. Antonucci N. (2014). The in vitro GcMAF effects on endocannabinoid system transcriptionomics, receptor formation, and cell activity of autism-derived macrophages. *J Neuroinflammation* 11:78. 10.1186/1742-2094-11-78 24739187 PMC3996516

[B66] Siniscalco D. Sapone A. Giordano C. Cirillo A. de Magistris L. Rossi F. (2013). Cannabinoid receptor type 2, but not type 1, is up-regulated in peripheral blood mononuclear cells of children affected by autistic disorders. *J. Autism. Dev. Disord.* 43 2686–2695. 10.1007/s10803-013-1824-9 23585028

[B67] Speed H. E. Masiulis I. Gibson J. R. Powell C. M. (2015). Increased cortical inhibition in autism-linked neuroligin-3R451C mice is due in part to loss of endocannabinoid signaling. *PLoS One* 10:e0140638. 10.1371/journal.,pone.0140638 26469287 PMC4607423

[B68] Suzuki K. Sugihara G. Ouchi Y. Nakamura K. Futatsubashi M. Takebayashi K. (2013). Microglial activation in young adults with autism spectrum disorder. *JAMA Psychiatry* 70 49–58. 10.1001/jamapsychiatry.2013.272 23404112

[B69] Tartaglione A. M. Schiavi S. Calamandrei G. Trezza V. (2019). Prenatal valproate in rodents as a tool to understand the neural underpinnings of social dysfunctions in autism spectrum disorder. *Neuropharmacology* 10.1016/j.expneurol.2017.04.017 [Epub ahead of print]. 30639388

[B70] Tumati S. Largent-Milnes T. M. Keresztes A. Ren J. Roeske W. R. Vanderah T. W. (2012). Repeated morphine treatment-mediated hyperalgesia, allodynia and spinal glial activation are blocked by co-administration of a selective cannabinoid receptor type-2 agonist. *J. Neuroimmunol.* 244 23–31. 10.1016/j.jneuroim.2011.12.021 22285397 PMC3298577

[B71] Turcotte C. Blanchet M. R. Laviolette M. Flamand N. (2016). The CB2 receptor and its role as a regulator of inflammation. *Cell. Mol. Life Sci.* 73 4449–4470. 10.1007/s00018-016-2300-4 27402121 PMC5075023

[B72] Vargas D. L. Nascimbene C. Krishnan C. Zimmerman A. W. Pardo C. A. (2005). Neuroglial activation and neuroinflammation in the brain of patients with autism. *Ann. Neurol.* 57 67–81. 10.1002/ana.20315 15546155

[B73] Vigli D. Cosentino L. Raggi C. Laviola G. Woolley-Roberts M. De Filippis B. (2018). Chronic treatment with the phytocannabinoid cannabidivarin (CBDV) rescues behavioural alterations and brain atrophy in a mouse model of Rett syndrome. *Neuropharmacology* 140 121–129. 10.1016/j.neuropharm.2018.07.029 30056123

[B74] Wei D. Dinh D. Lee D. Li D. Anguren A. MorenoSanz G. (2016). Enhancement of anandamide-mediated endocannabinoid signaling corrects autism-related social impairment. *Cannabis Cannabinoid Res.* 1 81–89. 10.1089/can.2015.0008 28861483 PMC5549436

[B75] Yeh M. L. Levine E. S. (2017). Perspectives on the role of endocannabinoids in autism spectrum disorders. *OBM Neurobiol.* 1:005. 10.21926/obm.neurobiol.1702005 PMC6407886 30854511

[B76] Zamberletti E. Beggiato S. Steardo L. Jr. Prini P. Antonelli T. Ferraro L. (2014). Alterations of prefrontal cortex GABAergic transmission in the complex psychotic-like phenotype induced by adolescent delta-9-tetrahydrocannabinol exposure in rats. *Neurobiol. Dis.* 63 35–47. 10.1016/j.nbd.2013.10.028 24200867

[B77] Zamberletti E. Gabaglio M. Parolaro D. (2017). The endocannabinoid system and autism spectrum disorders: insights from animal models. *Int. J. Mol. Sci.* 18:E1916. 10.3390/ijms18091916 28880200 PMC5618565

[B78] Zamberletti E. Gabaglio M. Piscitelli F. Brodie J. S. Woolley-Roberts M. Barbiero I. (2019). Cannabidivarin completely rescues cognitive deficits and delays neurological and motor defects in male Mecp2 mutant mice. *J. Psychopharmacol.* 33 894–907. 10.1177/0269881119844184 31084246

[B79] Zanelati T. V. Biojone C. Moreira F. A. Guimarães F. S. Joca S. R. (2010). Antidepressant-like effects of cannabidiol in mice: possible involvement of 5-HT1A receptors. *Br. J. Pharmacol.* 159 122–128. 10.1111/j.1476-5381.2009.00521.x 20002102 PMC2823358

[B80] Zoppi S. Madrigal J. L. Caso J. R. García-Gutiérrez M. S. Manzanares J. Leza J. C. (2014). Regulatory role of the cannabinoid CB2 receptor in stress-induced neuroinflammation in mice. *Br. J. Pharmacol.* 171 2814–2826. 10.1111/bph.12607 24467609 PMC4243857

[B81] Zuardi A. W. (2008). Cannabidiol: from an inactive cannabinoid to a drug with wide spectrum of action. *Rev. Bras. Psiquiatr.* 30 271–280. 10.1590/S1516-44462008000300015 18833429

