# Supplementary material for: Cannabidivarin Treatment Ameliorates Autism-Like Behaviors and Restores Hippocampal Endocannabinoid System and Glia Alterations Induced by Prenatal Valproic Acid Exposure in Rats
Source: Front Cell Neurosci. 2019 Aug 9;13:367. doi: 10.3389/fncel.2019.00367 (PMC6696797; doi:10.3389/fncel.2019.00367)
Supplement: Supplementary file 1 [file Data_Sheet_1.PDF]

Supplementary Material

1 Supplementary methods

1.1 Postnatal growth and maturation development in vehicle- and VPA-exposed rats

Weight gain in the offspring was measured at PND 9, 12, 15, 17 and 19. Eye opening was monitored once a day from PND 13 to 17 and scored as follows: 0=both eyes closed; 1=one eye open; and 2=both eyes open. At PND 9, nest-seeking behavior mediated by olfactory cues present in the home cage bedding was measured by individually placing the pups in the corner of a clean standard housing cage (425 x 266 x 185 mm) containing one plastic Petri dish (10 cm diameter) filled with home bedding on the opposite corner of the cage. The latency to reach the stimulus (both forepaws into the Petri dish) was measured with the aid of the Anymaze program (Ugo Basile, Italy). The cutoff time was 120 seconds. The cage was cleaned between animals with 0.1% acetic acid. To examine the pups' motor performance, each rat at PND 9 was placed on its back on a flat surface and the righting reflex was defined as the time required for each pup to return to its four limbs. The cutoff time was 30 seconds. Data were expressed as mean ± SEM of 28 vehicle- and 28 VPA-treated rats from 8 different litters and were analyzed by unpaired Student's t test.

2 Supplementary Results

Supplementary figure 1 shows the effect of VPA treatment on postnatal growth and maturation development in male pups. All vehicle- and VPA-treated dams gave birth at GD 21 and litter sizes were similar in the two experimental groups (vehicles: 12.667 ± 0.333; VPA: 13.500 ± 0.645; panel A). No postnatal mortality was observed and sex ratio in the litters from vehicle- and VPA-treated dams was similar (vehicles: males 53.5% ± 15.5, females 46.5% ± 15.5; VPA: males 45.5% ± 4.406, females 54.5% ± 4.406; panel B). Body weight was slightly but significantly reduced in VPA-exposed pups with respect to controls from PND 12 to 17 (panel C). Eye opening was delayed in VPA-exposed pups with respect to vehicles between PND 14 and 16 (panel D). Moreover, at PND 9, male pups from VPA-treated dams showed significantly increased latencies in nest-seeking behavior compared to controls (vehicles: 3.548 ± 2.255; VPA: 60.117 ± 12.859; panel E) as well as impaired righting reflex (vehicles: 3.001 ± 0.664; VPA: 6.340 ± 1.542; panel F).

3. Supplementary Figures

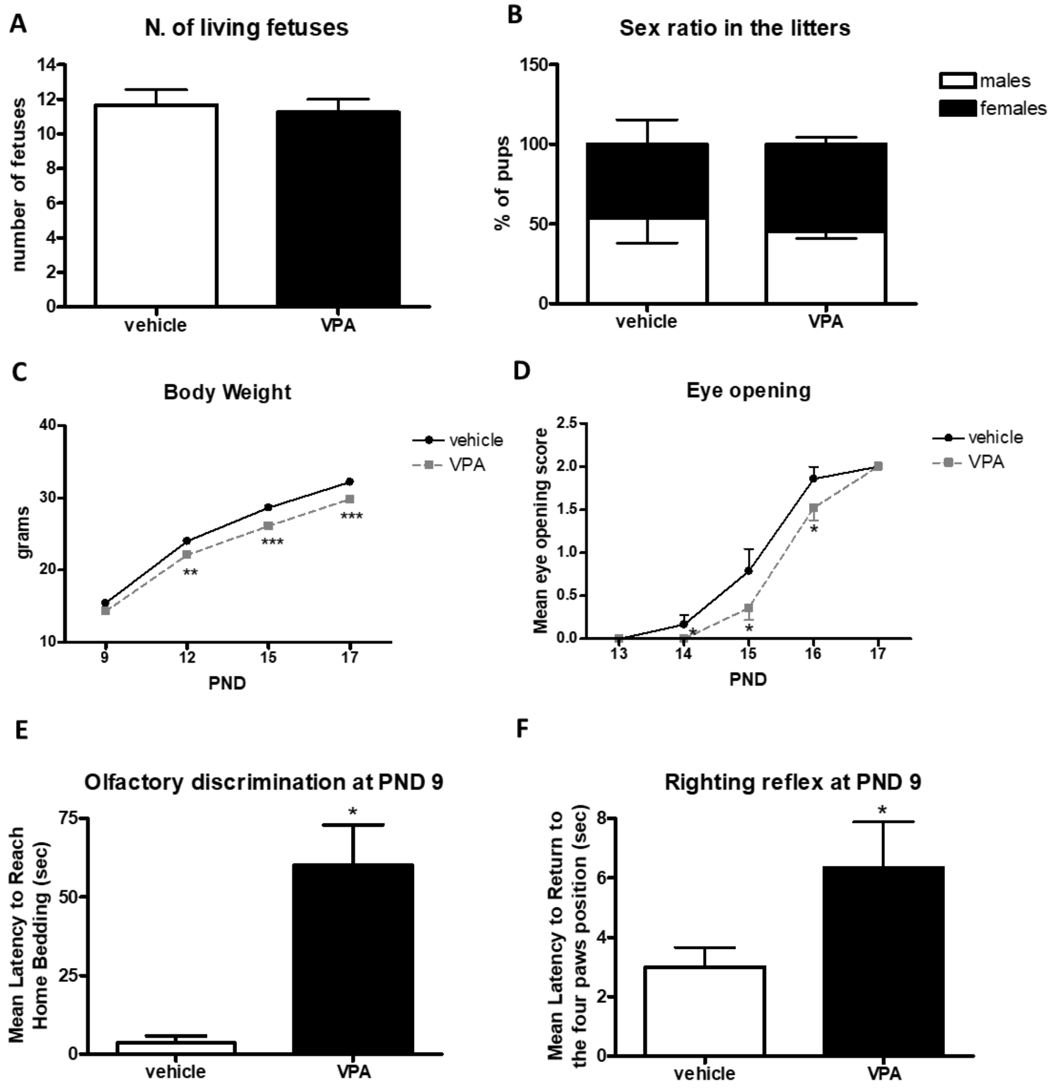

Supplementary Figure 1. Postnatal growth and maturation development in the male offspring from vehicle- and VPA-treated dams. Effect of a single injection of VPA (500 mg/kg i.p. at GD 12.5) on (A) litter sizes, (B) sex ratio in the litters, (C) body weight gain, (D) eye opening, as well as (E) olfactory discrimination and (F) righting reflex measured at PND 9 in the male offspring. Data represent mean ± SEM of 28 vehicle- and 28 VPA-treated rats from 8 different litters and were analyzed by unpaired Student's t test. ***p<0,001, **p<0,01, *p<0,05 vs vehicle.
